# Supplementary figures and images for: Dispersal patterns of Trypanosoma cruzi in Arequipa, Peru
Source: PLoS Negl Trop Dis. 2020 Mar 9;14(3):e0007910. doi: 10.1371/journal.pntd.0007910 (PMC7082062; doi:10.1371/journal.pntd.0007910)

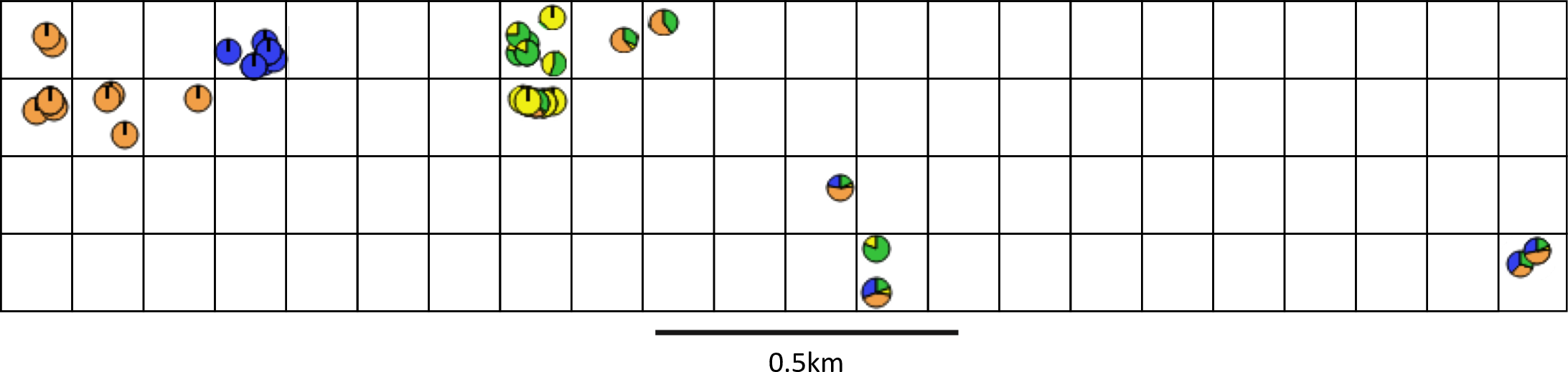

Supplement: S1 Fig — The colors of each pie chart represent the likelihood that a sample belongs to each of four genetic clusters. Each color (blue, yellow, orange, grey) represent a unique genetic cluster. There is significant clustering of identical genotypes within blocks 1–4, 5, and 7. Block 6 contains an exceptionally diverse population of T. cruzi. Block 8–12 have few samples per block, but the samples collected in each block are distinct from those collected elsewhere in the district. Blocks are displayed in a grid to maintain privacy. (TIF) [file pntd.0007910.s001.tif]

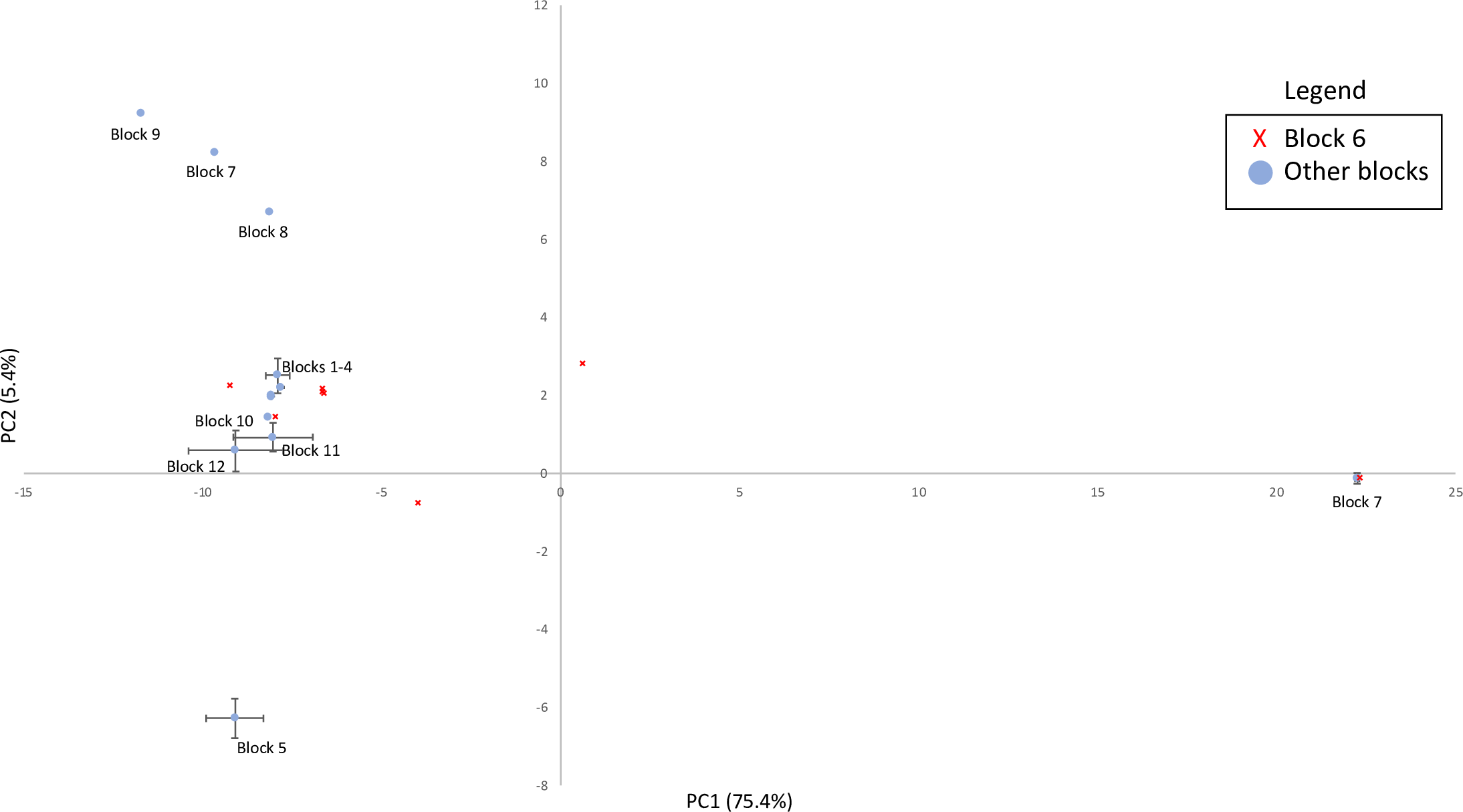

Supplement: S2 Fig — PCA was calculated using 56 samples. The centroids of all samples from each city block with standard error bars are shown here. Each of the seven samples collected in Block 6 are represented by a red X because they occupy disparate portions of the PCA space. One sample collected in Block 7 is unique and is thus represented by its own point. (TIF) [file pntd.0007910.s002.tif]

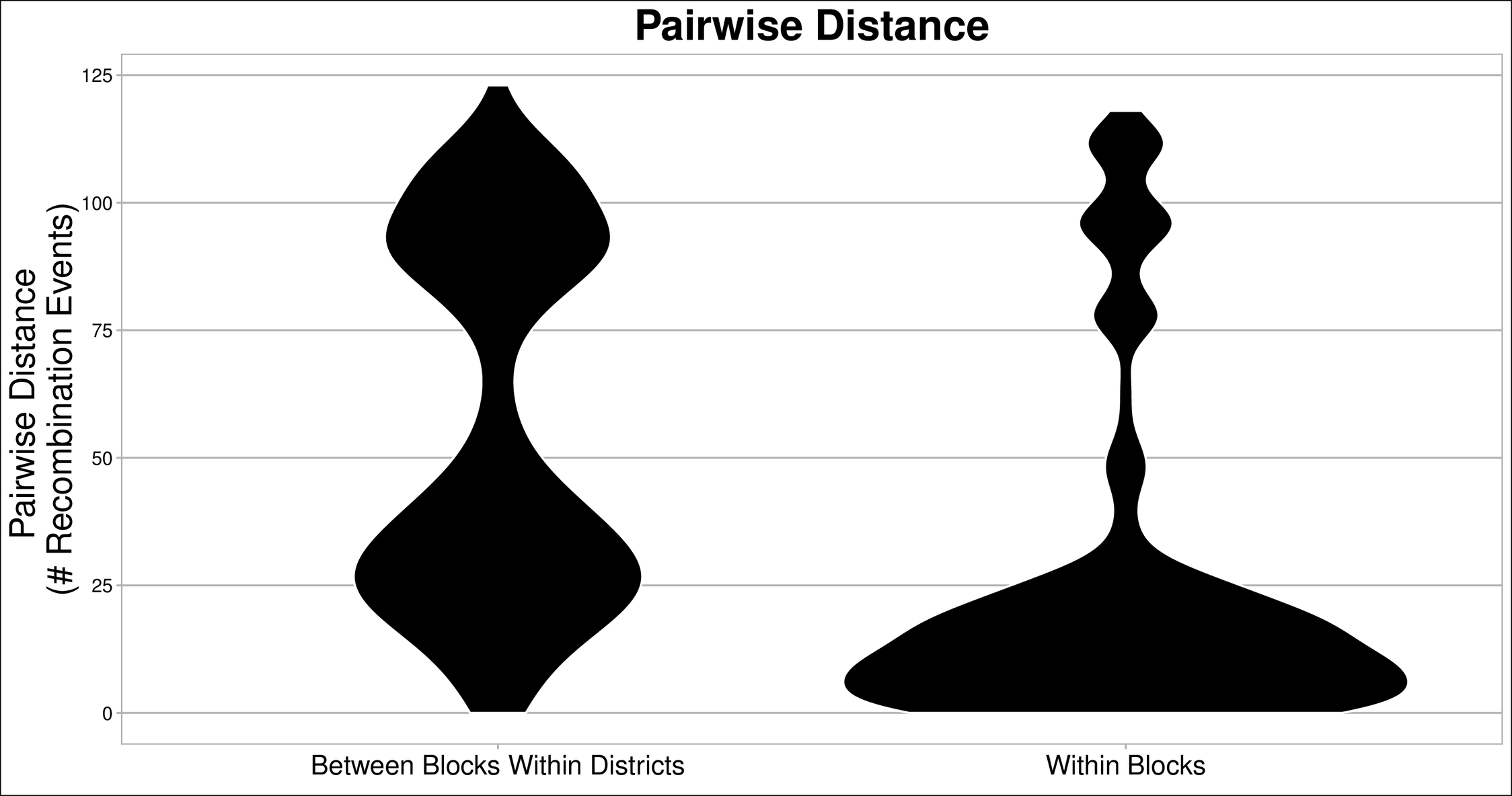

Supplement: S3 Fig — Violin plots show the number of pairwise differences in unique recombination events. Difference between all pairs of 123 samples, among pairs of samples collected in different blocks from the same district, and among pairs of samples collected in the same block, are shown. These results recapitulate the results found using genome-wide SNP data: The distribution of differences among samples between blocks among districts is larger than the distribution among all samples, the differences among samples within blocks are significantly smaller than expected given the diversity in the dataset (p<0.001). The results that genetically-similar samples cluster in blocks while the overall diversity is distributed among districts do not vary regardless of the data set analyzed (recombination events or genome-wide SNP data). (TIF) [file pntd.0007910.s003.tif]

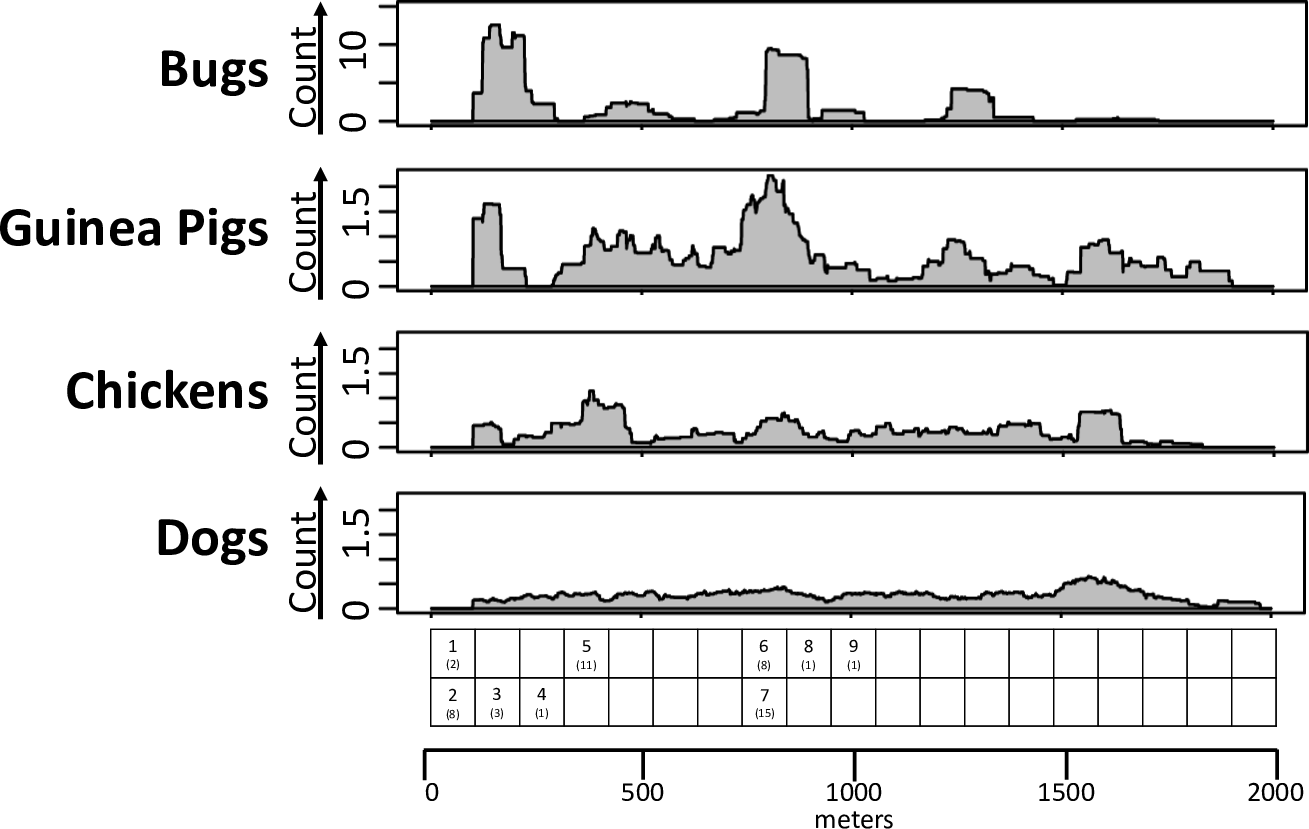

Supplement: S4 Fig — The average number of non-human hosts and T. infestans vectors was counted for each house along a 2km transect in Mariano Meglar. The distribution of guinea pigs along the transect is similar to the distribution of the vector. The number of dogs and chickens remains relatively constant across the transect and is not correlated with vector population densities. The y-axis depicts the average count of each species per house. Approximate location along the transect of numbered blocks from Fig 1 are shown. (TIF) [file pntd.0007910.s004.tif]

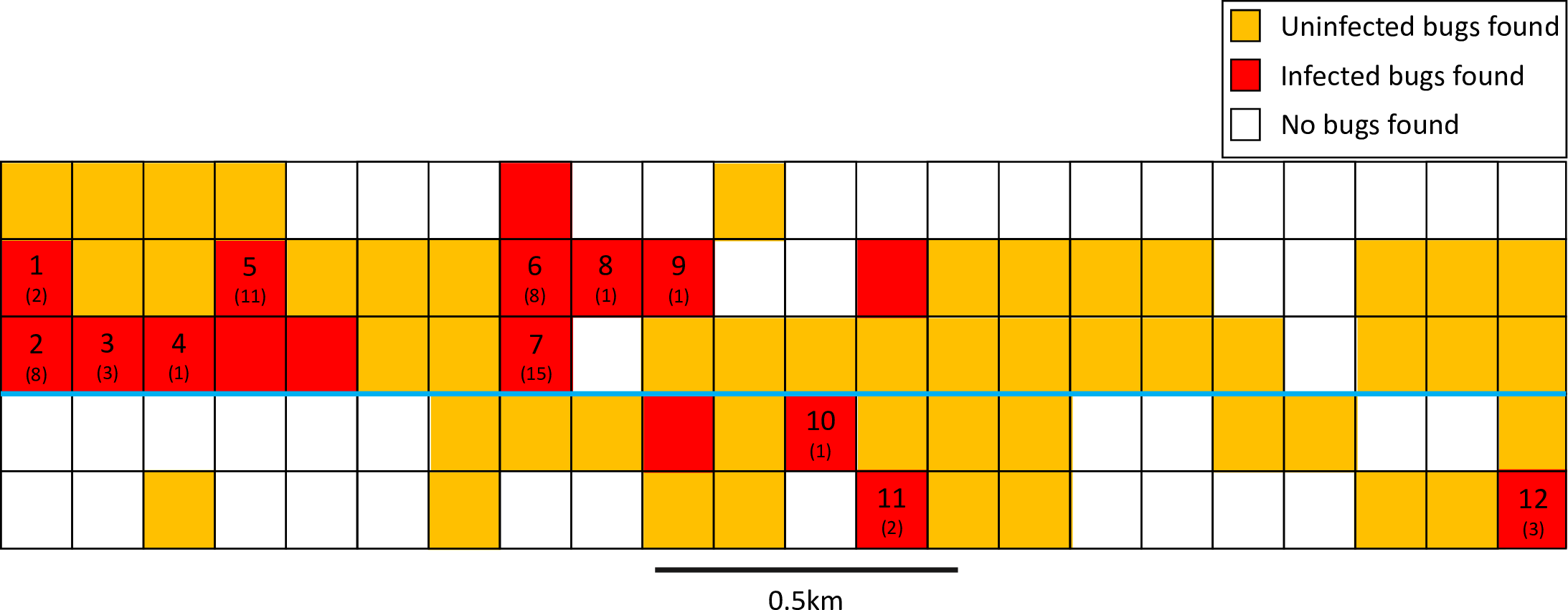

Supplement: S5 Fig — Blocks containing infected vectors are colored red, blocks where only uninfected vectors were found are colored orange, and blocks where no vectors were detected are colored white [60]. Blocks where an infected vector was found but the parasite genome was not sequenced here are represented by red blocks without block numbers. Blocks above the blue line were meticulously surveyed for parasites at four timepoints. Blocks below the blue line were surveyed by the Ministry of Health. T. cruzi-infected vectors were found in three additional blocks in Mariano Melgar outside of this region. (TIF) [file pntd.0007910.s005.tif]

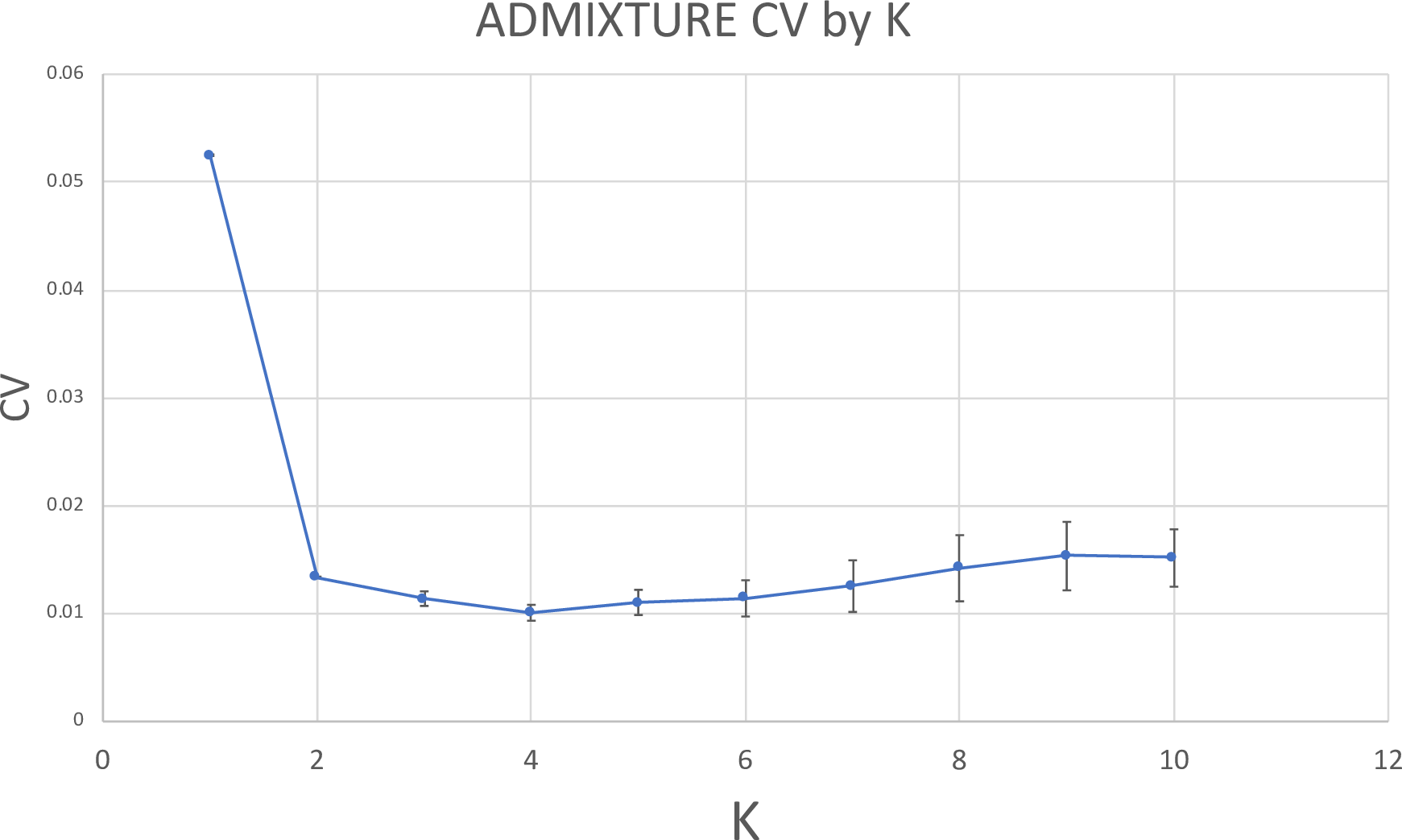

Supplement: S6 Fig — Standard error bars are shown for each value. K = 4 was determined to be the optimal number of genetic clusters. (TIF) [file pntd.0007910.s006.tif]
