# Supplementary material for: Dispersal patterns of Trypanosoma cruzi in Arequipa, Peru
Source: PLoS Negl Trop Dis. 2020 Mar 9;14(3):e0007910. doi: 10.1371/journal.pntd.0007910 (PMC7082062; doi:10.1371/journal.pntd.0007910)
Supplement: S1 Table — (DOCX) [file pntd.0007910.s007.docx]

| *Test* | *P-value* | *% total variation* |
| --- | --- | --- |
| *Variation within blocks* | 0.001 | 37.131922 |
| *Variation among blocks within districts* | 0.001 | 62.5097975 |
| *Variations between districts* | 0.477 | 0.3582805 |
| *Variation within blocks in Mariano Melgar* | N/A | 34.94804 |
| *Variation between blocks in Mariano Melgar* | 0.001 | 65.05196 |
