# Supplementary material for: Dispersal patterns of Trypanosoma cruzi in Arequipa, Peru
Source: PLoS Negl Trop Dis. 2020 Mar 9;14(3):e0007910. doi: 10.1371/journal.pntd.0007910 (PMC7082062; doi:10.1371/journal.pntd.0007910)
Supplement: S2 Table — (DOCX) [file pntd.0007910.s008.docx]

| **Sample ID** | **District** | **Block** | **House** | **Ecotype^*^** | **Year Collected** | **Source** | **SRA Accession Number** |
| --- | --- | --- | --- | --- | --- | --- | --- |
| TC126 | Bustamante | 1 | 1 | Yard | 2012 | *T. infestans* | SRS4304931 |
| TC125 | Bustamante | 1 | 2 | Corral | 2012 | *T. infestans* | SRS4304930 |
| TC119 | Cayma | 1 | 1 | Bedroom | 2011 | *T. infestans* | SRS4304925 |
| TC122 | Cayma | 2 | 1 | Bedroom | 2011 | *T. infestans* | SRS4304927 |
| TC143 | Huanca | N/A | 1 | Bedroom | 2015 | *T. infestans* | SRS4304885 |
| TC141 | Huanca | N/A | 2 | Bedroom | 2015 | *T. infestans* | SRS4304887 |
| TC145 | Huanca | N/A | 2 | Bedroom | 2015 | *T. infestans* | SRS4304883 |
| TC148 | Huanca | N/A | 2 | Corral | 2015 | *T. infestans* | SRS4304907 |
| TC146 | Huanca | N/A | 2 | Yard | 2015 | *T. infestans* | SRS4304890 |
| TC149 | Huanca | N/A | 3 | Corral | 2015 | *T. infestans* | SRS4304906 |
| TC151 | Huanca | N/A | 4 | Bedroom | 2015 | *T. infestans* | SRS4304908 |
| TC142 | Huanca | N/A | 5 | Yard | 2015 | *T. infestans* | SRS4304884 |
| TC150 | Huanca | N/A | 6 | Bedroom | 2015 | *T. infestans* | SRS4304909 |
| TC152 | Huanca | N/A | 6 | Corral | 2015 | *T. infestans* | SRS4304903 |
| TC144 | Huanca | N/A | 7 | Yard | 2015 | *T. infestans* | SRS4304882 |
| TC135 | Huanca | N/A | 8 | Yard | 2013 | *T. infestans* | SRS4304983 |
| TC131 | La Joya | 1 | 1 | Corral | 2013 | *T. infestans* | SRS4304987 |
| TC040 | La Joya | 1 | 1 | Room 1 | 2008 | *T. infestans* | SRS4304948 |
| TC129 | La Joya | 1 | 1 | Room 1 | 2013 | *T. infestans* | SRS4304989 |
| TC130 | La Joya | 1 | 1 | Room 2 | 2013 | *T. infestans* | SRS4304988 |
| TC155 | La Joya | 1 | 1 | Room 2 | 2015 | *T. infestans* | SRS4304904 |
| TC147 | La Joya | 1 | 1 | Yard | 2015 | *T. infestans* | SRS4304891 |
| TC022 | La Joya | 2 | 1 | N/A | 2008 | *T. infestans* | SRS4304956 |
| TC014 | La Joya | 3 | 1 | Yard | 2008 | *T. infestans* | SRS4304936 |
| TC001 | La Joya | 4 | 1 | Corral 1 | 2008 | *T. infestans* | SRS4304935 |
| TC002 | La Joya | 4 | 1 | Corral 1 | 2008 | *T. infestans* | SRS4304934 |
| TC020 | La Joya | 4 | 1 | N/A | 2008 | *T. infestans* | SRS4304958 |
| TC016 | La Joya | 4 | 2 | Room | 2008 | *T. infestans* | SRS4304940 |
| TC026 | La Joya | 4 | 3 | Room | 2008 | *T. infestans* | SRS4304954 |
| TC003 | La Joya | 5 | 1 | Bedroom | 2008 | *T. infestans* | SRS4304939 |
| TC033 | La Joya | 6 | 1 | Room | 2008 | *T. infestans* | SRS4304960 |
| TC004 | La Joya | 6 | 2 | Yard | 2008 | *T. infestans* | SRS4304938 |
| TC029 | La Joya | 6 | 2 | Yard | 2008 | *T. infestans* | SRS4304952 |
| TC037 | La Joya | 6 | 3 | Corral | 2008 | *T. infestans* | SRS4304945 |
| TC019 | La Joya | 6 | 4 | N/A | 2008 | *T. infestans* | SRS4304959 |
| TC027 | La Joya | 6 | 5 | Corral | 2008 | *T. infestans* | SRS4304953 |
| TC015 | La Joya | 7 | 1 | Yard | 2008 | *T. infestans* | SRS4304941 |
| TC034 | La Joya | 8 | 1 | Room | 2008 | *T. infestans* | SRS4304946 |
| TC038 | La Joya | 9 | 1 | Corral | 2008 | *T. infestans* | SRS4304950 |
| TC031 | La Joya | 9 | 2 | Corral | 2008 | *T. infestans* | SRS4304961 |
| TC023 | La Joya | 9 | 3 | Corral | 2008 | Guinea pig | SRS4304955 |
| TC035 | La Joya | 9 | 4 | Corral | 2008 | *T. infestans* | SRS4304947 |
| TC036 | La Joya | 9 | 5 | Room | 2008 | *T. infestans* | SRS4304944 |
| TC039 | La Joya | 9 | 6 | Corral | 2008 | *T. infestans* | SRS4304951 |
| TC115 | Mariano Melgar | 1 | 1 | Corral | 2011 | *T. infestans* | SRS4304923 |
| TC071 | Mariano Melgar | 1 | 2 | Yard | 2011 | *T. infestans* | SRS4304866 |
| TC073 | Mariano Melgar | 2 | 1 | Yard | 2011 | *T. infestans* | SRS4304864 |
| TC042 | Mariano Melgar | 2 | 2 | Corral 1 | 2010 | Guinea pig | SRS4304943 |
| TC043 | Mariano Melgar | 2 | 2 | Corral 1 | 2010 | Guinea pig | SRS4304914 |
| TC045 | Mariano Melgar | 2 | 2 | Corral 1 | 2010 | Guinea pig | SRS4304919 |
| TC070 | Mariano Melgar | 2 | 2 | Corral 1 | 2011 | *T. infestans* | SRS4304869 |
| TC061 | Mariano Melgar | 2 | 2 | Room | 2010 | Guinea pig | SRS4304874 |
| TC075 | Mariano Melgar | 2 | 2 | Yard | 2011 | *T. infestans* | SRS4304862 |
| TC079 | Mariano Melgar | 2 | 3 | Yard | 2011 | *T. infestans* | SRS4304854 |
| TC074 | Mariano Melgar | 3 | 1 | Yard | 2011 | *T. infestans* | SRS4304865 |
| TC072 | Mariano Melgar | 3 | 2 | Yard | 2011 | *T. infestans* | SRS4304867 |
| TC105 | Mariano Melgar | 3 | 3 | Corral | 2011 | *T. infestans* | SRS4304969 |
| TC064 | Mariano Melgar | 4 | 1 | Corral | 2010 | Guinea pig | SRS4304878 |
| TC010 | Mariano Melgar | 5 | 1 | Roof | 2008 | *T. infestans* | SRS4304937 |
| TC111 | Mariano Melgar | 5 | 2 | Patio | 2011 | *T. infestans* | SRS4304964 |
| TC078 | Mariano Melgar | 5 | 3 | Corral | 2011 | *T. infestans* | SRS4304871 |
| TC049 | Mariano Melgar | 5 | 3 | Room | 2010 | *T. infestans* | SRS4304913 |
| TC076 | Mariano Melgar | 5 | 3 | Yard | 2011 | *T. infestans* | SRS4304863 |
| TC077 | Mariano Melgar | 5 | 3 | Yard | 2011 | *T. infestans* | SRS4304870 |
| TC041 | Mariano Melgar | 5 | 4 | Room | 2010 | Dog | SRS4304942 |
| TC116 | Mariano Melgar | 5 | 5 | Yard | 2011 | *T. infestans* | SRS4304924 |
| TC080 | Mariano Melgar | 5 | 6 | Room | 2011 | *T. infestans* | SRS4304853 |
| TC104 | Mariano Melgar | 5 | 6 | Yard | 2011 | *T. infestans* | SRS4304970 |
| TC112 | Mariano Melgar | 5 | 6 | Yard | 2011 | *T. infestans* | SRS4304963 |
| TC088 | Mariano Melgar | 6 | 1 | N/A | 2011 | *T. infestans* | SRS4304859 |
| TC107 | Mariano Melgar | 6 | 2 | Yard | 2011 | *T. infestans* | SRS4304967 |
| TC099 | Mariano Melgar | 6 | 3 | Roof | 2011 | *T. infestans* | SRS4304980 |
| TC090 | Mariano Melgar | 6 | 4 | Stacked Stone | 2011 | *T. infestans* | SRS4304972 |
| TC097 | Mariano Melgar | 6 | 5 | Corral | 2011 | *T. infestans* | SRS4304976 |
| TC044 | Mariano Melgar | 6 | 6 | Corral | 2010 | Dog | SRS4304916 |
| TC055 | Mariano Melgar | 6 | 6 | Room | 2010 | Guinea pig | SRS4304894 |
| TC102 | Mariano Melgar | 6 | 6 | Yard | 2011 | *T. infestans* | SRS4304979 |
| TC048 | Mariano Melgar | 7 | 1 | Corral | 2010 | *T. infestans* | SRS4304920 |
| TC081 | Mariano Melgar | 7 | 2 | Corral 1 | 2011 | *T. infestans* | SRS4304856 |
| TC103 | Mariano Melgar | 7 | 2 | Corral 2 | 2011 | *T. infestans* | SRS4304971 |
| TC085 | Mariano Melgar | 7 | 2 | Corral 3 | 2011 | *T. infestans* | SRS4304852 |
| TC082 | Mariano Melgar | 7 | 2 | Room 1 | 2011 | *T. infestans* | SRS4304855 |
| TC089 | Mariano Melgar | 7 | 2 | Room 2 | 2011 | *T. infestans* | SRS4304858 |
| TC084 | Mariano Melgar | 7 | 2 | Yard | 2011 | *T. infestans* | SRS4304849 |
| TC086 | Mariano Melgar | 7 | 2 | Yard | 2011 | *T. infestans* | SRS4304851 |
| TC083 | Mariano Melgar | 7 | 3 | Corral 1 | 2011 | *T. infestans* | SRS4304850 |
| TC047 | Mariano Melgar | 7 | 3 | Corral 2 | 2010 | *T. infestans* | SRS4304921 |
| TC124 | Mariano Melgar | 7 | 3 | Room | 2012 | *T. infestans* | SRS4304929 |
| TC065 | Mariano Melgar | 7 | 4 | Yard | 2011 | *T. infestans* | SRS4304860 |
| TC091 | Mariano Melgar | 7 | 4 | Yard | 2011 | *T. infestans* | SRS4304973 |
| TC092 | Mariano Melgar | 7 | 4 | Yard | 2011 | *T. infestans* | SRS4304974 |
| TC046 | Mariano Melgar | 7 | 5 | Corral | 2010 | *T. infestans* | SRS4304918 |
| TC098 | Mariano Melgar | 8 | 1 | Bathroom | 2011 | *T. infestans* | SRS4304977 |
| TC100 | Mariano Melgar | 9 | 1 | Yard | 2011 | *T. infestans* | SRS4304861 |
| TC110 | Mariano Melgar | 10 | 1 | Workshop | 2011 | *T. infestans* | SRS4304965 |
| TC108 | Mariano Melgar | 11 | 1 | Corral | 2011 | *T. infestans* | SRS4304966 |
| TC101 | Mariano Melgar | 11 | 2 | Bedroom | 2011 | *T. infestans* | SRS4304978 |
| TC068 | Mariano Melgar | 12 | 1 | Corral | 2011 | *T. infestans* | SRS4304881 |
| TC069 | Mariano Melgar | 12 | 1 | Room | 2011 | *T. infestans* | SRS4304868 |
| TC095 | Mariano Melgar | 12 | 2 | Roof | 2011 | *T. infestans* | SRS4304975 |
| TC051 | Miraflores | 1 | 1 | Near Corral | 2010 | *T. infestans* | SRS4304898 |
| TC134 | Miraflores | 2 | 1 | Room | 2013 | *T. infestans* | SRS4304984 |
| TC133 | Miraflores | 2 | 2 | Bedroom | 2013 | *T. infestans* | SRS4304985 |
| TC132 | Miraflores | 3 | 1 | Bedroom | 2013 | *T. infestans* | SRS4304986 |
| TC137 | Miraflores | 4 | 1 | Yard | 2014 | *T. infestans* | SRS4304981 |
| TC136 | Miraflores | 4 | 2 | Corral | 2014 | *T. infestans* | SRS4304982 |
| TC123 | Sachaca | 1 | 1 | Corral | 2012 | *T. infestans* | SRS4304928 |
| TC106 | Sachaca | 1 | 2 | Room | 2011 | *T. infestans* | SRS4304968 |
| TC067 | Sachaca | 1 | 2 | Yard | 2011 | *T. infestans* | SRS4304857 |
| TC113 | Sachaca | 2 | 1 | Patio | 2011 | *T. infestans* | SRS4304962 |
| TC114 | Sachaca | 2 | 1 | Yard | 2011 | *T. infestans* | SRS4304922 |
| TC053 | Tiabaya | 1 | 1 | Room | 2010 | *T. infestans* | SRS4304900 |
| TC058 | Tiabaya | 1 | 2 | Corral | 2010 | *T. infestans* | SRS4304897 |
| TC057 | Tiabaya | 1 | 3 | Corral | 2010 | *T. infestans* | SRS4304896 |
| TC140 | Tiabaya | 2 | 1 | Yard | 2015 | *T. infestans* | SRS4304886 |
| TC154 | Tiabaya | 3 | 1 | Corral | 2015 | *T. infestans* | SRS4304905 |
| TC156 | Tiabaya | 3 | 1 | Yard | 2015 | *T. infestans* | SRS4304911 |
| TC153 | Tiabaya | 3 | 2 | Corral | 2015 | *T. infestans* | SRS4304902 |
| TC139 | Tiabaya | 4 | 1 | Room | 2015 | *T. infestans* | SRS4304889 |
| TC127 | Tiabaya | 5 | 1 | Room | 2012 | *T. infestans* | SRS4304990 |
| TC063 | Uchumayo | 1 | 1 | Bedroom | 2010 | *T. infestans* | SRS4304879 |
| TC120 | Uchumayo | 1 | 1 | Room | 2011 | *T. infestans* | SRS4304926 |
| TC138 | Vitor | 1 | 1 | N/A | 2013 | *T. infestans* | SRS4304888 |

^*^ ‘Corral’ ecotype = guinea pig corral
