# Supplementary material for: Dispersal patterns of Trypanosoma cruzi in Arequipa, Peru
Source: PLoS Negl Trop Dis. 2020 Mar 9;14(3):e0007910. doi: 10.1371/journal.pntd.0007910 (PMC7082062; doi:10.1371/journal.pntd.0007910)
Supplement: Attachment — Submitted filename: migration_paper_reviewer_comments_responses.docx [file pntd.0007910.s010.docx]

Thank you for your time and effort on this manuscript. We have revised the manuscript following the recommendations of the reviewers. The comments from the reviewers are written in Helvetica font and our responses in Calibri font following the marker “>>”.

Editors:

Thank you very much for submitting your manuscript "Dispersal patterns of Trypanosoma cruzi in Arequipa, Peru" (#PNTD-D-19-01885) for review by PLOS Neglected Tropical Diseases. Your manuscript was fully evaluated at the editorial level and by independent peer reviewers. The reviewers appreciated the attention to an important problem, but raised some substantial concerns about the manuscript as it currently stands. As detailed below, these concerns are mainly referred to the sampling and analysis of the data. In addition to the reviewers' comments, we would like to stress that it is known that even within 'restricted' domiciliary settings the presence of distinct parasite genotypes circulating in different hosts (i.e. humans, dogs, triatomines) has been described, which may contribute to the overall genotypic variability. Therefore, it should be considered (at least discussed) that sampling mostly triatomines may have biased somehow the results. These issues must be addressed before we would be willing to consider a revised version of your study. We cannot, of course, promise publication at that time.

>>The authors agree with the editors that the sampling scheme is relevant to the results of this study and the manuscript would be improved with greater detail. We have dedicated a new paragraph discussing the advantages and potential caveats of our sampling scheme in regards to the present study in the Discussion.

Line 292: “Most *T. cruzi* sampled here were isolated from the only vector species in Arequipa, *T. infestans*, which reduces dataset biases for specific *T. cruzi* genotypes as *T. cruzi* from all infected mammal hosts must be transmitted to *T. infestans* to complete its life cycle. The *T. cruzi* samples were isolated from dogs and guinea pigs support this hypothesis as these samples are closely related to those isolated from vectors. Further, because *T. cruzi* must pass through *T. infestans* to complete its life cycle, the parasite must infect a vector in order to proliferate in a new environment. Previous studies in domestic settings have found that the vector population harbors a more diverse *T. cruzi* population than hosts, suggesting that vectors carry the strains in local hosts in addition to strains not yet transmitted from vector to host (Llewellyn *et al*. 2009). One potential caveat is that *T. cruzi* genotypes residing long-term in mammalian hosts that have not transmitted to bugs will remained unsampled. Indeed, the only *T. cruzi* isolated from a human in this dataset has a unique genotype from those sequenced from bugs, dogs, and guinea pigs (Berry *et al*. 2019). However, epidemiological models suggest that infected hosts are likely to transmit *T. cruzi* to vectors given sufficient time and vector abundance. That is, one infected human infects 21 vectors on average while 100 vectors on average are needed to infect one human (Fabrizio *et al.* 2016; Flores-Ferrer *et al.* 2019). By sampling *T. cruzi* primarily from vectors we likely capture the least biased representation of the diversity in the city in order to determine how a single population of *T. cruzi* migrated through Arequipa.”

Reviewer #1:

In this study, Brisson et al use high resolution genomic tools (multilocus SNP) to survey the dispersal patterns of Trypanosoma cruzi among different districts of Arequipa (Peru). The metholology is sound and the conclusions are convincing.
The paper may be accepted as it is. However, the following points might be considered: 

1. it would have been informative to characterize the isolates of the present study by comparison with the general classification of T. cruzi genotypes (DTU TcI-VI). It can be guessed that all Arequipa isolates are TcI. However, the adding of a few reference strains would have been informative.

>> We thank the reviewer for pointing out this omission. All isolates are indeed TC1, and this information has been added to the text.

Line 86: “123 DTU 1 *T. cruzi* parasites, representing a single, closely related population [3], were collected from ten geographically-distinct districts throughout the region of Arequipa, Peru (Fig 1).”

2. The relative genetic uniformity of T. cruzi genotypes of the present study clashes with former studies in which the presence of highly distinct DTUs has been evidenced in Arequipa (Brenière et al., 1985, Isoenzymic studies and epidemiological data of Trypanosoma cruzi from Arequipa (Peru), Pacific side. Ann. Soc. belge méd. trop. 65 (suppl.1): 63-66).

>> Thank you for pointing to this prior paper. Brenière et al 1985 isotyped 39 samples from a broad region in southern Peru, of which 35 represented the same isoenzymic strain. Our data appear to confirm these prior studies, suggesting that *T. cruzi* in the Arequipa region have very low genetic variability. An interesting additional note is that diversity appears low in the entire region, as many samples in Brenière et al were from >100km outside of Arequipa, compared to our study which includes primarily samples from the city of Arequipa itself. In fact, the only distinct *T. cruzi* genotype identified in Brenière et al was collected in a valley far from Arequipa. We hypothesize that there may be several *T. cruzi* strains present among bugs in the valleys surrounding Arequipa, but that the extant genotype in the city itself descended from a single founder (Berry *et al.* 2019).

Line 290: “The *T. cruzi* genotypes sampled in Arequipa are exceptionally uniform, as discussed previously (Berry *et al.* 2019), and as shown in previous reports of the broader region surrounding Arequipa (Breniere *et al.* 1985).”

3. Isn't it possible to have an idea about the age of the most common ancestor (MCA) of these lineages? For example, using similar methodologies, yhe origin of the Escherichia coli ST131 clone has been dated a little bit before 2000 ("measurably evolving pathogens") (Stoesser N, Sheppard AE, Pankhurst L, De Maio N, Moore CE, Sebra R, et al. (2016) Evolutionary History of the Global Emergence of the Escherichia coli Epidemic Clone ST131. mBio 7: eo2162-15.). Such dating would be quite relevant in the present study.

>> We agree with the reviewer that adding divergence time estimates can be important for contextualizing results. We estimated divergence time in a previous publication to be incredible recent. It is important to note, however, that the estimates have large associated errors due to inaccurate root time estimates and an inconsistent evolutionary clock. We added a statement in the results indicating the estimate for the MRCA.

Line 92: The common ancestor of the Arequipan *T. cruzi* population was estimated to live as recently as 8.8kya, however there is significant uncertainty in estimates of divergence times [3].

4. Typo page 17 line 14: "hwoever".

>> We thank the reviewer for pointing out this mistake. This has been fixed (Line 283).

Reviewer #2:

In this manuscript the authors describe the genetic diversity of Trypanosoma cruzi, the etiological agent of Chagas disease by using in depth sequencing. This is an interesting manuscript describing the distribution of genotypes at different scales in Arequipa. The results are original and clearly within the scope of the journal. There are however some major points that should in my opinion be addressed by the authors before the manuscript can be accepted for publication.

1. Can we really speak of dispersal of T. cruzi? Is it not a distribution pattern analyses? 

>>We characterized timing and rates of gene flow using classical population genetics methods to assess migration among geographic locations. These methods, including AMOVA, ADMIXTURE, and associated population genetics methods, are the current standard for estimating migration and have considerable theoretical and empirical validation. It is important to note that our analyses are not distribution pattern analyses but statistical analyses of geospatially-tagged genome sequences. These methods are designed and vetted to assess gene flow, which is mediated by dispersal, among areas.

2. As no human-related isolate has been analyzed in this study, I think this should be clarified in the title, in the abstract and throughout the discussion of the manuscript.

>>It is important to note that this study was designed to assess dispersal of a microbe across geographic space over time. By sampling primarily from vectors, we are able to estimate patterns of dispersal across space for a less biased sample of the *T. cruzi* population, regardless of the inclusion of isolates from particular species. Although related, we did not intend to assess human incidence patterns caused by infection of this microbe. Many prior articles report that human *T. cruzi* infections are rare and do not impact parasite dispersal suggesting that isolates from vectors provide the best dataset with which to estimate parasite dispersal. We have made the aim of this manuscript and the associated sampling scheme more clear in the abstract and within the manuscript. We feel that changing the title could mislead readers into thinking we estimated dispersal among individuals rather than across space.

Line 27: “We used population genomic and phylogenomic tools to analyze whole genomes of 123 *T. cruzi* isolates derived from vectors and non-human mammals throughout Arequipa to determine patterns of *T. cruzi* dispersal.”

Line 292: “Most *T. cruzi* sampled here were isolated from the only vector species in Arequipa, *T. infestans*, which reduces dataset biases for specific *T. cruzi* genotypes as *T. cruzi* from all infected mammal hosts must be transmitted to *T. infestans* to complete its life cycle. The *T. cruzi* samples were isolated from dogs and guinea pigs support this hypothesis as these samples are closely related to those isolated from vectors. Further, because *T. cruzi* must pass through *T. infestans* to complete its life cycle, the parasite must infect a vector in order to proliferate in a new environment. Previous studies in domestic settings have found that the vector population harbors a more diverse *T. cruzi* population than hosts, suggesting that vectors carry the strains in local hosts in addition to strains not yet transmitted from vector to host (Llewellyn *et al*. 2009). One potential caveat is that *T. cruzi* genotypes residing long-term in mammalian hosts that have not transmitted to bugs will remained unsampled. Indeed, the only *T. cruzi* isolated from a human in this dataset has a unique genotype from those sequenced from bugs, dogs, and guinea pigs (Berry *et al*. 2019). However, epidemiological models suggest that infected hosts are likely to transmit *T. cruzi* to vectors given sufficient time and vector abundance. That is, one infected human infects 21 vectors on average while 100 vectors on average are needed to infect one human (Fabrizio *et al.* 2016; Flores-Ferrer *et al.* 2019). By sampling *T. cruzi* primarily from vectors we likely capture the least biased representation of the diversity in the city in order to determine how a single population of *T. cruzi* migrated through Arequipa.”

3. My main concern refers to the origin of the samples which is not clear to me. Please indicate how many bugs from how many houses originated the 123 samples employed. I would suggest including in Suppl Table2 the host from which each sample was isolated, and the ecotope in the house where the bugs were collected from. Also, I would suggest ordering the information of this table by district, block and house (and not by district only as it is now) (i.e.: District, Block number, house number, host, number of samples).

>>The authors thank the reviewer for this great suggestion. S2 Table has been updated to include House #, Ecotype, and vector/host species, which provides significantly more detail regarding sample collection.

Line 638: “**S2 Table. Sample collection location, source, and year.”**

4. Albeit I'm not a geneticist, or perhaps because of that, I have some doubts regarding the genetic distances informed: In Results section (First paragraph). How can the average pairwise genetic distance between all samples be smaller (1726 SNPs) than the average pairwise distance between samples from different blocks within each district (1925 SNPs)?

>>The average pairwise distance among all samples is smaller than the average pairwise distance among samples from different blocks because of the similarity among samples from the same block. This is, in fact, one of the major conclusions of these analyses. Excluding comparisons of these very similar samples within blocks results in the greater average pairwise distance seen in the between-block comparisons. For example, imagine two blocks where a value (say height) was measured for two individuals per block:

block 1 = (2,1); 2 = (10,9). Average pairwise differences among all samples = (2-1 + 10-2 + 10-1 + 9-2 +9-1 + 10-9 )/6 where 6 is the number of comparisons = 1+8+9+7+8+1 = 34/6 = 5.667. If you remove the WITHIN BLOCK comparisons, there are 4 total comparisons = 8+9+7+8 = 32/4 = 8. In this example, the average pairwise difference overall (5.667) is less than the average pairwise difference in the between blocks comparison (8).

5. In Results section (First paragraph of page 9) Can the limited genetic diversity within each block be due to a small number of houses sampled in each block? 
6. In Results section (First paragraph of page 10) I wonder if the observed pattern could be due to the fact that one or few houses have been sampled rather than “a block” effect. It is not clear to me if the effect of different houses within a block can be assessed with this sampling design.

>> We apologize for the confusion. We have now made clear the number of houses sampled per block and the number of samples sequenced per block. While it is true that only one *T. cruzi* was found and sequenced on some blocks, multiple isolates were sequenced on many of the blocks (20 of the 37 sampled).

Line 88: “Multiple isolates were sequenced from 20 of the 37 blocks in Arequipa where *T. cruzi* were found. On only three of these blocks were all of the isolates sequenced cultured from different bugs collected from a single house, although the isolates were collected in different years in two of these cases. On 17 blocks, an average of 5 isolates were sequenced which were cultured from bugs collected from an average of 3 houses per block.”

The interesting result is that we frequently find clones within the same block, and almost never in neighboring and distant blocks. It is also important to note that if only one sample was sequenced on a block, no within block comparison was performed and thus these blocks do not affect the within-block genetic distance values given. Importantly, the transect in Mariano Melgar was sampled extensively, such that an effort was made to sample every house. We added a sentence to make the sampling effort more clear.

Line 339: “An effort was made to inspect every house along this transect and collect bugs whenever possible such that *T. cruzi* isolates from most houses that harbored infected bugs were sequenced (S5 Fig).”

7. Regarding Figure 3’s legend. Is it not expected that districts that are more deeply (i.e Mariano Melgar and La Joya) sample exhibit a higher genetic diversity? 

>>Deeper sampling certainly increases the chances of capturing a greater proportion of the diversity. Mariano Melgar and La Joya were the most deeply sampled areas yet very little diversity was detected, suggesting little diversity exists. Additionally, the positive correlation between diversity and sampling does not hold in our dataset. For example, Miraflores has much greater detected diversity than Tiabaya despite having far fewer genomes sampled. It is also important to note that the analyses performed are not comparing diversity among districts but exist to demonstrate that much of the diversity within each district can also be found in other districts. That many very closely related isolates can be found in geographically separated districts suggests significant between-district migration. We re-phrased the Figure 3 legend to make this more clear.

Line 137: “*T. cruzi* from each district, for example, Mariano Melgar and La Joya, span the phylogenetic diversity in the region suggesting that most of the genetic variation in Arequipa can be found within each single district.”

8. Results section after the tables, page 12. Authors state “T. cruzi collected from different blocks tend to be unrelated regardless of spatial proximity (Fig 2)” Based on which analyses have the authors based this statement?  I do not agree Fig 2 illustrates this statement. Also Supl. Fig 2 seems to show that the very distinct genotypes from Block 6 and Block 7 were very near.

>>We agree with the reviewer that this statement was too definitive given the evidence and have softened the language in the revised manuscript. The main conclusion, that *T. cruzi* found in the same block are often very closely related while those in different blocks are typically much more distantly related, remains strongly supported by the data and analyses.

Line 153: “*T. cruzi* collected from the same block tend to be very closely related compared to parasites found in different blocks (Fig 2).”

9. Have you analyzed if there are any differences due to the year of sample collection? 

>>This is a very good question. There does not appear to be a year effect, although only in Mariano Melgar are the same houses sampled at multiple times, so year is unfortunately correlated with space. However, blocks 1-4 are sampled over time and are still very similar, suggesting that the spatial effects overwhelm any temporal effects.

Line 107: “… although sampling is limited, we find no evidence of temporal variation.”

10. Discussion-First paragraph. “The genomic data support a pattern of migration in which dispersal proceeds with little impediment among houses within a city block soon after colonization of the block (Fig. 2 and S1).”  How do the authors know that “soon after colonization”? I believe the authors have not done any temporal analyses. 

>> We thank the reviewer for this feedback. Several surveys of this region address the rate of the within-block dispersal processes. For example, Levy *et al.* 2014 showed that the *T. cruzi* population in Block 5 was not founded until 2010, and by 2011 infected bugs were collected in multiple houses throughout the block. Our data show that this population was indeed descended from a single founder. We have added a section to describe the temporal statement.

Line 196: “The genomic data support the previously described pattern of migration in which dispersal proceeds with little impediment among houses within a city block soon after colonization of the block (Figs 2 and S1) [5]. For example, the *T. cruzi* population on Block 5 was founded by a single lineage in 2010 and could be collected from multiple houses throughout the block by 2011 (Fig 3) [5].”

Line 241: “… the recently established population in Block 5 [5] has not invaded neighboring blocks (Fig 3). These data suggest that migration to adjacent blocks is rare but can occur given sufficient time, supporting the hypothesis of a low inter-block dispersal rate.”

11. Discussion-Page 15 Second paragraph. Could the observed pattern be explained just by a recent colonization of the block (not by real barriers) and with time and transmission occurrence other neighboring blocks be colonized as in blocks 1-4?

>> This is a good point. The limited between-block dispersal, compared with the abundance of within-block dispersal, suggests that there are greater barriers to between than within-block migration given that both could be occurring at the same time. We do hypothesize that migration between blocks does occur, and is more likely to occur given sufficient time. Compared to within-block migration, however, between-block migration is significantly more rare. For example, the population in blocks 1-4 is an older population (Levy *et al.* 2014) such that it has had sufficient time for the population to expand to neighboring blocks. By contrast, the recently-founded population currently found in many houses within Block 5 suggests that within-block migration occurs much more readily than between-block migration. The large population in Block 7 also supports this hypothesis, however as it is an older population than Block 5, a single close relative of the Block 7 population was found across the street in Block 6.

Line 238: “Nevertheless, dispersal of both *T. cruzi* and *T. infestans* has occurred between neighboring blocks, especially in older areas of the city. For example, a single *T. cruzi* lineage colonized neighboring Blocks 1-4 and a sample collected in Block 6 appears to have migrated from Block 7 (Fig 3). By contrast, the recently established population in Block 5 [5] has not invaded neighboring blocks (Fig 3). These data suggest that migration to adjacent blocks is relatively rare but can occur given sufficient time, supporting the hypothesis of a low inter-block dispersal rate.”

12. Line numbers are missing.

>> We apologize for this frustrating omission and have fixed this for these revisions.

13. I was not able to distinguish the colors in Figure 3. Could making lines thicker improve this figure?

>> We agree with the reviewer that this figure could be improved for clarity and have modified it.

14. Please include axes legends of Supl Fig 4.

>>This is a great suggestion and we have made the changes in the new SFig 4.

15. M&M Please spell out VCF (in ref to VCF files).

>>We thank the reviewer for pointing out this omission and have spelled out the acronym.

Line 354: “Individual genomic Variant Call Format (gVCF) files containing SNP data for each sample were generated using GATK HaplotypeCaller”

16. Discussion. Page 14. “Interestingly, geographic proximity to blocks with an active population increases the establishment probability of that block only slightly (Figs 3 and S1)” What do authors consider “Active population” ? Please clarify.

>>We agree with the reviewer that this phrasing is confusing and have rephrased the section to make it clear that we are simply referring to blocks containing a population of *T. cruzi*.

Line 203: “Interestingly, geographic proximity to blocks harboring a *T. cruzi* population increases the establishment probability of that block only slightly (Figs 3 and S1).”

17. Please revise Table 2 legend. It includes, in fact among district data.

>>We agree with the reviewer that this title was poorly-written and have made it more clear.

Line 150: “**Table 2. There is significant population genetic structure among blocks within districts, but not among districts.”**
